# Efficient non-cytotoxic fluorescent staining of halophiles

**DOI:** 10.1038/s41598-018-20839-7

**Published:** 2018-02-07

**Authors:** Ivan Maslov, Andrey Bogorodskiy, Alexey Mishin, Ivan Okhrimenko, Ivan Gushchin, Sergei Kalenov, Norbert A. Dencher, Christoph Fahlke, Georg Büldt, Valentin Gordeliy, Thomas Gensch, Valentin Borshchevskiy

**Affiliations:** 1 Research Center for Molecular Mechanisms of Aging and Age-Related Diseases, Moscow Institute of Physics and Technology, 141700 Dolgoprudniy, Russia; 2 Mendeleyev University of Chemical Technology of Russia, 125047 Moscow, Russia; 3 CSI Organic Chemistry and Biochemistry, Technische Universität Darmstadt, 64287 Darmstadt, Germany; 4 Institute of Complex Systems (ICS), ICS-4: Cellular Biophysics, Forschungszentrum Jülich GmbH, 52428 Jülich, Germany; 5 Univ. Grenoble Alpes, CEA, CNRS, IBS, 38000 Grenoble, France; 6 Institute of Complex Systems (ICS), ICS-6: Structural Biochemistry, Forschungszentrum Jülich GmbH, 52425 Jülich, Germany

## Abstract

Research on halophilic microorganisms is important due to their relation to fundamental questions of survival of living organisms in a hostile environment. Here we introduce a novel method to stain halophiles with MitoTracker fluorescent dyes in their growth medium. The method is based on membrane-potential sensitive dyes, which were originally used to label mitochondria in eukaryotic cells. We demonstrate that these fluorescent dyes provide high staining efficiency and are beneficial for multi-staining purposes due to the spectral range covered (from orange to deep red). In contrast with other fluorescent dyes used so far, MitoTracker does not affect growth rate, and remains in cells after several washing steps and several generations in cell culture. The suggested dyes were tested on three archaeal (*Hbt. salinarum, Haloferax sp., Halorubrum sp*.) and two bacterial (*Salicola sp., Halomonas sp*.) strains of halophilic microorganisms. The new staining approach provides new insights into biology of *Hbt. salinarum*. We demonstrated the interconversion of rod-shaped cells of *Hbt. salinarium* to spheroplasts and submicron-sized spheres, as well as the cytoplasmic integrity of giant rod *Hbt. salinarum* species. By expanding the variety of tools available for halophile detection, MitoTracker dyes overcome long-standing limitations in fluorescence microscopy studies of halophiles.

## Introduction

Halophiles are organisms that can thrive in extreme conditions of high salt concentrations. Most of these organisms belong to the archaea or bacteria life domains. Exposed to a hostile environment, halophiles evolved unique biomechanisms, which make them very exciting scientific objects. Probably, the most important is the example of bacteriorhodopsin – a photoactivatable retinal membrane protein with unique properties, including stability at high concentration of salt (up to 5M), protease resistance, tolerance to high temperatures (upto 140 °C when dry)^1^ and to a broad range of pH (at least 3–10)^2^. Bacteriorhodopsin has become the most studied membrane protein, often serving as a “model” for all other membrane proteins (see for instance^3–8^).

In recent decades, halophiles have attracted additional scientific interest due to their isolation from Earth’s subsurface halites, in some cases >250 million years old^9–20^. Being buried in brine-filled fluid inclusions, halophiles are able to survive for millennia under conditions of extremely high ionic strength, elevated temperatures and nutrients depletion. Genome studies showed that spore formation is not possible for halophilic archaea^21^, thus, their survival mechanisms remain unclear^22^.

Halites were discovered on the surface of Mars by the *Opportunity* and *Spirit* rovers and in meteorite samples^23–29^. It was shown that they can hypothetically play protective role against radiation^30^ or biochemical degradation^19^ and potentially harbour halobacteria^31^. Thus, sensitive detection of living halophiles in hypersaline medium with minimal influence on their viability is a question of great interest for further exploration of microbial life on Earth and elsewhere.

An additional interest associated with halophiles arises from their role in microbial communities of natural and man-made hypersaline environments^32^, especially in the case of hydrocarbonoclastic communities, which are potentially applicable for bioremediation of oil pollutions^33^.

Fluorescent staining can provide a sensitive method for detection of halophilic species (i.e. via flow cytometry) in environmental samples, and can also be used for their analysis via fluorescence microscopy. The main challenge in relation to the fluorescent staining of halophiles is the high ionic strength of the medium and in some cases hostile pH^34^ of their natural environment, which are inappropriate for conventional antibodies and dyes^35^. The low permeability of the halobacterial S-layer to macromolecules can additionally complicate immunostaining.

The list of staining approaches for halophiles is currently rather limited. Most of them require fixation (i.e. immunostaining^36,37^, DAPI^38,39^, and FISH^38,40^) or the use of DNA-targeted intercalating dyes (i.e. LIVE&DEAD kit^35,39,41–45^, Acridine Orange^43,46^, Hoechst^47^). The latter approach is unsafe due to interference with the cellular DNA processing machinery, which can result in complete loss of cell viability (for review see^48,49^). It has been shown that Acridine Orange and its derivatives can additionally stain archaeal phospholipids^50^. Several other approaches for staining halophiles that have been recently suggested^44,51^ remain limited to particular applications. Genetic modification of archaea with GFP for its fluorescent visualization^44,52^ is also worth mentioning, although it is not straightforward for detection purposes. Notably, no dyes for long-term staining without cytotoxic side-effects have been found; the field is dominated by DNA-staining dyes, which has cytotoxicity as an intrinsic limitation.

Here we suggest a new method for staining living halobacteria cells with fluorescent dyes in their growth medium. We show that dyes designed to specifically stain mitochondria in mammalian eukaryotic cells (MitoTracker Orange CMTMRos, MitoTracker Red CMXRos and MitoTracker Deep Red FM) easily permeate the S-layer of *Hbt. salinarum* and cause stable bright staining of the archaeon. Even without removal of the residual, non-incorporated MitoTracker dye, the signal-to-noise ratio (SNR) is sufficient for clear cell detection and fluorescence microscopy. To test our staining procedure we investigate the conversion of *Hbt. salinarum* cells into spheroplasts via exposure to an EDTA-containing solution. In the case of MitoTracker Orange CMTMRos we also show that it does not have an effect on the cell’s growth rate. The staining remains bright during long observations (hours to days) and is inherited in cell division. The new method of staining allowed us to detect the development of viable spheres of submicron size (herein called “microspheres” to highlight the difference between them and spheroplasts) from rod-shaped *Hbt. salinarum* after the exposure to EDTA, which was previously assumed to result only in spheroplast formation. In addition, we demonstrate the cytosolic integrity of giant *Hbt. salinarum* cells via fluorescence loss in the photobleaching (FLIP) approach. In addition, the dyes were successfully applied to three halophilic microorganisms isolated from environmental samples from hypersaline lakes representing both the bacterial and archaeal domains of life. Therefore, we propose that MitoTracker dyes can be used as an effective and non-harmful dyes for halophile staining.

## Materials and Methods

### Cell culture

The *Hbt. salinarum* strain S9 was used as test sample for halophilic microorganism. The growth medium contained 250 g of NaCl, 20 g of MgSO_4_·7H_2_O, 3 g of Na_3_C_6_H_5_O_7_·2H_2_O, 2 g of KCl and 10 g of peptone (Helicon, Russia, H-1906-0.5) (per 1 litre), at pH 6.5. Cells were routinely cultured in 100 mL flasks at 37 °C with shaking (200 rpm).

### Sample preparation and staining

MitoTracker Green FM (M7514), MitoTracker Orange-CMTMRos (M7510), MitoTracker Red-CMXRos (M7512) and MitoTracker Deep Red FM (M22426) (Thermo Fisher Scientific, USA) were dissolved in DMSO to obtain 1 mM stock solutions. Solutions were stored at −20 °C.

**Figure 1:**
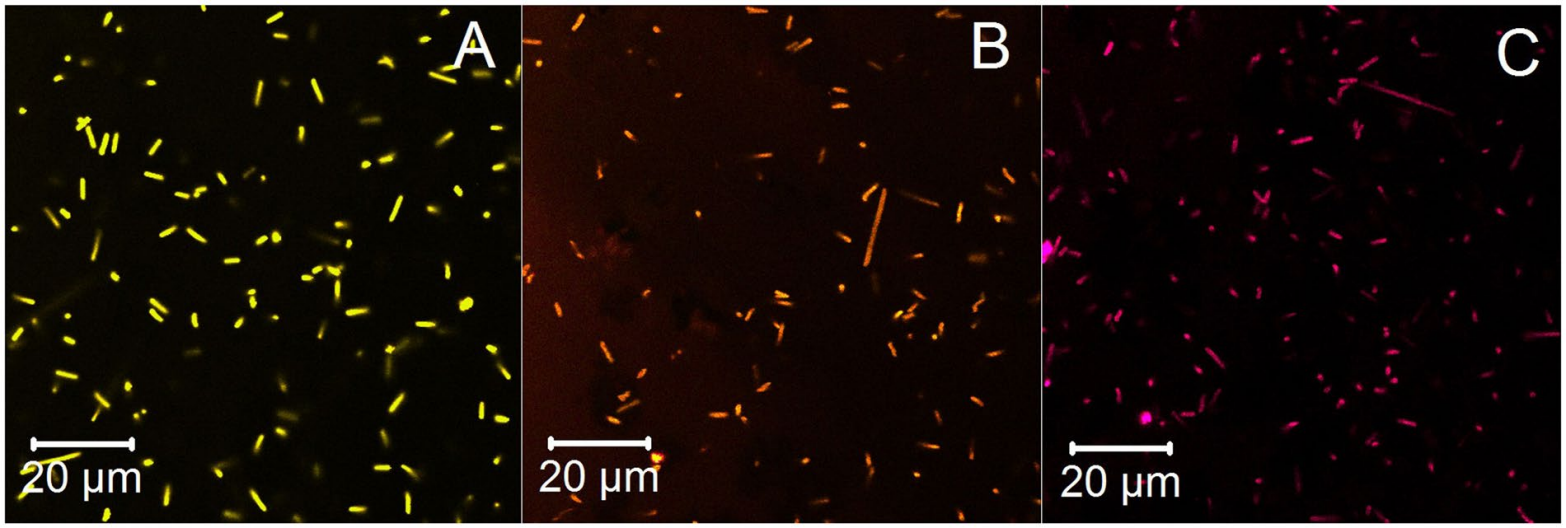
Living *Hbt. salinarum* cells in growth medium stained with MitoTracker Orange CMTMRos (**A**), MitoTracker Red CMXRos (**B**) and MitoTracker Deep Red FM (**C**).

To stain *Hbt. salinarum* (Fig. 1) or species isolated from environmental samples, MitoTracker solution was added to the growth medium at a 1:1000 dilution (to a final concentration of 1 μM).

### Confocal fluorescence microscopy

Measurements were performed in thin-bottom 8-well chambered cover glass (Thermo Fisher Scientific, USA, 155409), using an apochromate objective (420792-9800-720, Zeiss) with oil immersion (ISO 8036, Immersol 518F, Zeiss).

MitoTrackers were excited by 561 nm laser for MitoTrackers Orange-CMTMRos and Red-CMXRos, and by 633 nm laser for Deep Red FM. Emission was recorded in λ-mode with 34-channel QUASAR detector unit from Zeiss. All images were processed using Zen 2012 (Zeiss, Germany) and Fiji^53^ software. Supplementary Video 2 was drift corrected using a StackReg -plugin for Fiji^54^.

### Growth rate measurements

*Hbt. salinarum* cells were diluted in growth medium to an optical density of approximately 0.05 at 600 nm (OD_600nm_) (with growth medium used for blank).  5 mL cell cultures were incubated in 15 mL flasks for a week at 37 °C with shaking (200 rpm).

**Figure 2:**
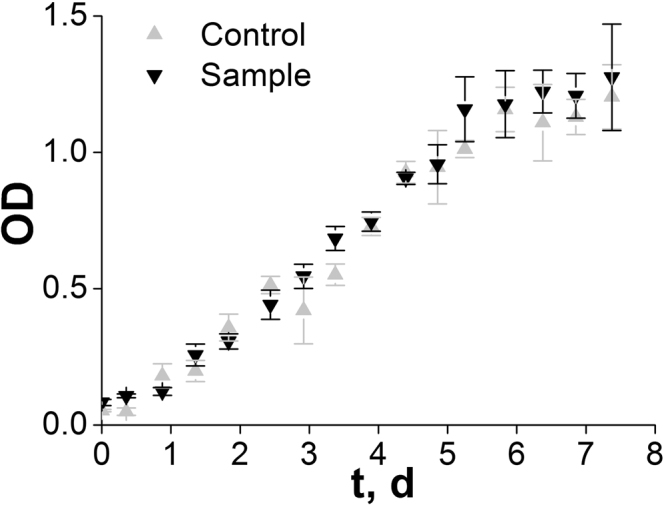
Growth curves of stained and control cells of *Hbt. salinarum*. The cells were grown with (sample group; downward-pointing triangles) and without (control group; upward-pointing triangles) MitoTracker Orange CMTMRos.

MitoTracker Orange CMTMRos was added twice a day to the first flask (sample), to keep the ratio between number of bacteria and MitoTracker molecules approximately constant and to maintain conditions similar to those in the fluorescence microscopy experiments during the entire observation period, namely, 1 μM of dye per OD_600nm_ unit. No MitoTracker Orange CMTMRos was added to the second flask, so it could serve as a control. The OD_600nm_ was measured for eight days in each flask. Growth medium with and without MitoTracker were used as a reference for sample and control. Aliquots used for the OD_600nm_ determination were returned to the respective flask after each measurement. The growth curves in Fig. 2 represent the average of 3 independent samples.

### MitoTracker durability during cultivation

**Figure 3:**
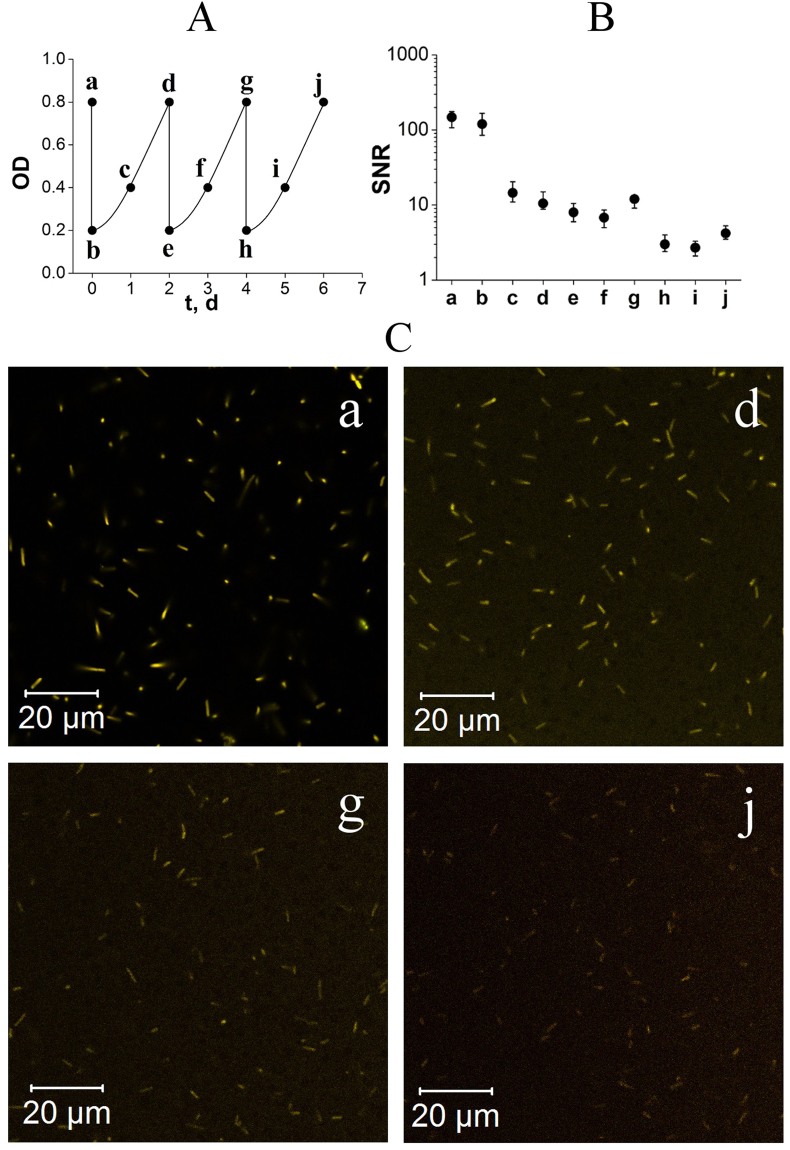
Durability of MitoTracker staining during several growth and dilution cycles. (**A**) Schematic plot of OD_600nm_ change during the experiment: 2-day exponential growth periods correspond to incubation time, while a 4-fold drop corresponds to the dilution. Particular time-points are labelled with lower-case Latin letters (a–j). (**B**) SNR decrease in the experiment. (**C**) (a) Microphotograph of cell culture at the day one; (d) two days after staining; (g) four days after staining and (j) six days after staining. Lower-case Latin letters in B and C correspond to the particular measurement time, shown in (**A**). Halobacteria were stained with MitoTracker Orange CMTMRos. The dye was washed out via centrifugation after staining. Cell cultures were incubated in a dye-free growth medium for 6 days with dilution every 2 days to maintain the active growth phase. The intensity of excitation laser was the same for all images. The detector gain was equal for images *d,g,j*, but it was reduced for image *a* to avoid saturation. The variation of the background in each image is due to different distances from the focal plane to the surface of the cover glass. Contrast stretching was applied to achieve the highest contrast of each image.

*Hbt. salinarum* cells were stained with MitoTracker Orange CMTMRos and after a 24-h incubation in a dye-containing medium (time point “a” in Fig. 3), the cells were sedimented via centrifugation (at 2000 g 37 °C for 5 min) and resuspended into MitoTracker-free growth medium to a final OD_600nm_ of 0.2 (“b”, Fig. 3). Cells were cultured at growth conditions for two days (to OD_600nm_ of 0.8; “d”, Fig. 3) and then diluted again to an OD_600nm_ of 0.2 (time point “e”, Fig. 3). This way, cells always remained in the exponential growth phase. Each day a sample of the cell culture was taken and a fluorescence image was acquired. The dilution-growth cycle was repeated three times. The plot of OD_600nm_ as a function of time from the start of the experiment as well as images obtained using fluorescence microscopy of the samples are shown in Fig. 3. Images were analyzed to estimate SNR. SNR was calculated for 20 cells for each time point as a ratio of difference in the mean signal in the cells and outside divided by the standard deviation of the signal outside the cells (see Fig. 3B).

### Conversion to spheroplasts

**Figure 4:**
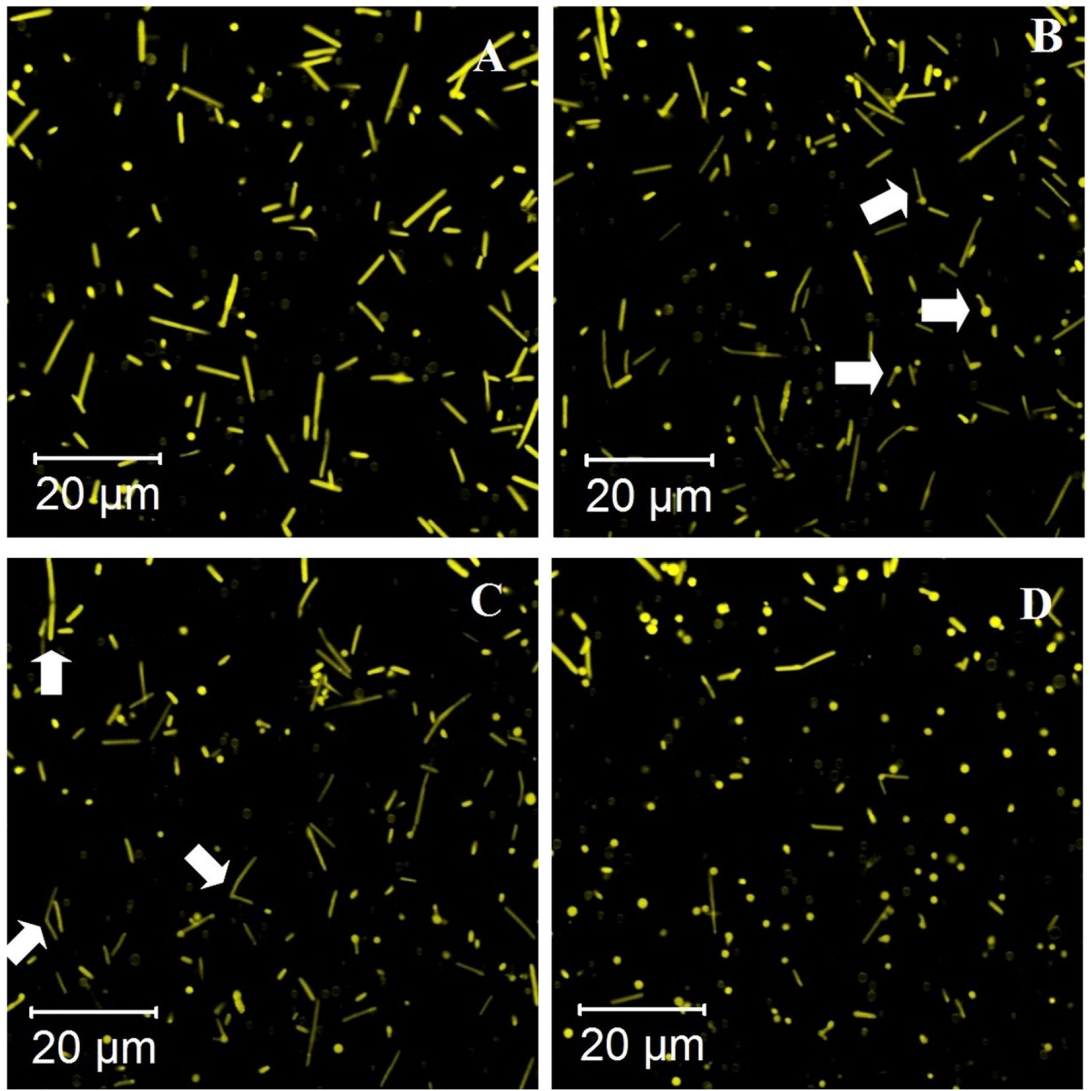
Conversion of *Hbt. salinarum* cells stained with MitoTracker Orange CMTMRos into spheroplasts by EDTA treatment. The entire process lasts approximately 10 min: in the beginning the cells have a clear rod shape (**A**; t = 0 s). During the conversion, the short cells swell at one end (**B**, arrows; t = 200 s), while long cells first bend around their midpoint (**C**, arrows; t = 260 s). Finally, both types of cells form spheroplasts (**D**, t = 480 s). Cells with intermediate shapes (swollen or bent) are indicated with arrows.

Spheroplasts were prepared as previously described^46^. Following 15 min of incubation in a staining solution cells in a 2 mL sample of culture (OD_600nm_ 0.8–1.0) were sedimented via centrifugation (at 2000 g 37 °C for 5 min). The supernatant was removed by pipetting. Cells were resuspended in 150 μL spheroplast-forming solution (2 M NaCl, 27 mM KCl, 50 mM Tris hydrochloride pH 8.75, 15% sucrose, 15% glycerol) and transfered to one well of the thin-bottom 8-well chambered cover glass (Thermo Fisher Scientific, USA, 155409). The excitation laser was focused slightly above the bottom of the cover glass. The first measurement (time point 0) was recorded immediately after 15 μL of 0.5 M EDTA was added into the well. The obtained data are shown in Fig. 4 and Supplementary Video 1.

**Figure 5:**
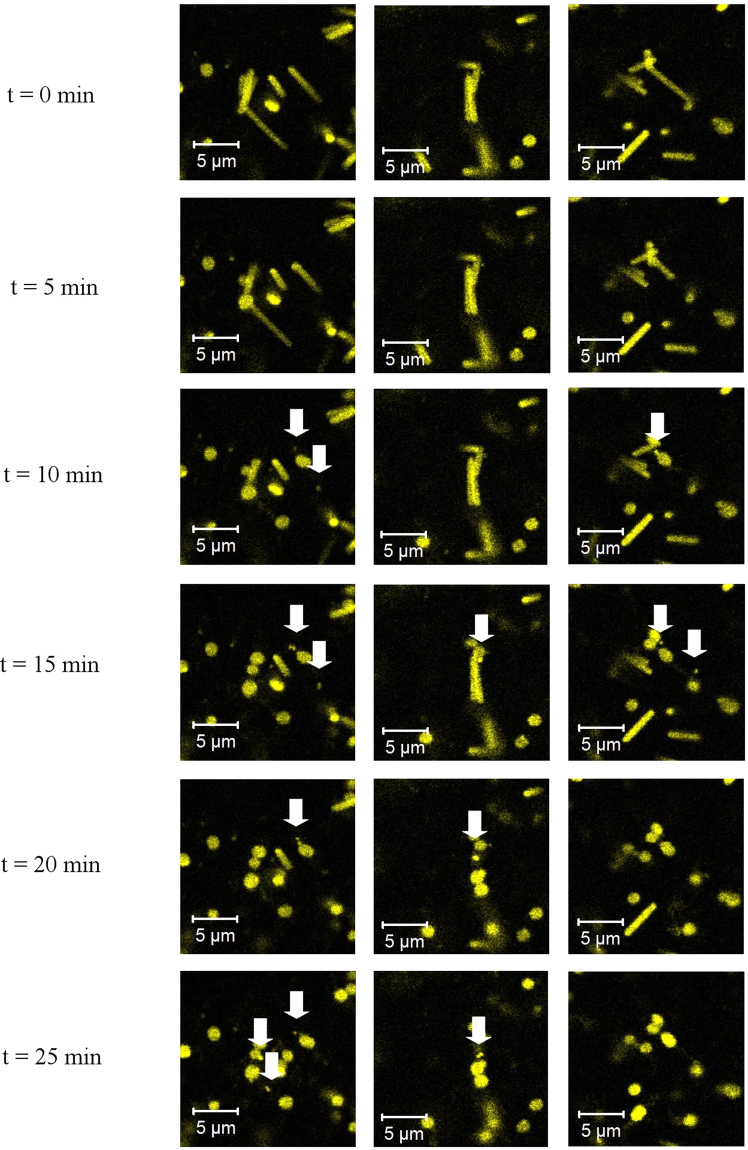
Appearance of microspheres during conversion of *Hbt. salinarum* cells into spheroplasts by EDTA treatment. *Hbt. salinarum* cells were stained with MitoTracker Orange CMTMRos, entrapped in 2% agar and exposed to EDTA in a spheroplast-forming solution, as described in Materials and Methods. In addition to the spheroplasts formation, the generation of microspheres (marked by arrows) was observed. In contrast to rod-shaped cells and spheroplasts, microspheres are mobile in agar, but fluctuate only in proximity of spheroplasts originating from the same rod-shaped cells. This can be explained by fluctuation inside cavities formed in the agar after the contraction of the rod-shaped cells.

For a more detailed observation of the conversion process, the *Hbt. salinarum* cells were stained with MitoTracker Orange CMTMRos. Cells were concentrated by sedimentation (4000 g for 10 min at room temperature) and entrapped in a 2% agar solution (Helicon, Russia, H0102) with growth medium. The mixture was distributed on the bottom of microscopy dish to form a thin layer. After solidification the gel was washed 3 times with a spheroplast forming solution and then observed by fluorescence microscopy for up to 10 hours in an excess of spheroplast forming solution containing 50 mM EDTA (see Fig. 5 and Supplementary Video 2).

### Recovery of rod-shaped *Hbt. salinarum* from spheroplasts and microspheres

*Hbt. salinarum* cells were stained with MitoTracker Orange CMTMRos in the growth medium and then converted to spheroplasts as described above. Microspheres were isolated from the mixture by filtration through a 0.45-µm PTFE filter (4555, Pall Corporation, Russia). Sample purity was confirmed by fluorescent microscopy (see Supplementary Figure 2C). In total, 500 µL of sample containing filtered microspheres was mixed with 2 mL of rich growth medium (250 g/L NaCl, 2 g/L MgSO_4_·7H_2_O, 3 g/L KCl, 3 g/L Na_3_C_6_H_5_O_7_, 0.2 g/L CaCl_2_, 5.0 g/L tryptone (Serva, Germany, 48647.01), 2.0 g/L yeast extract (Organotechnie, France, 19512), 1.25 g/L glycerol, 1 mg/L ZnSO_4_·7H_2_O, 50 mg/L FeSO_4_·7H_2_O, 0.3 mg/L MnSO_4_·H_2_O, pH 7.0.) with 15% sucrose and incubated at 37 °C for 10 days in the dark.

Spheroplasts were isolated from the microsphere-containing mixture by centrifugation in a spheroplast forming solution at 20000 g for 20 min at room temperature. At these conditions, spheroplasts were dominantly sedimented, while microspheres remained in the supernatant. Centrifugation was repeated 4 times. Each time, the supernatant was discarded and the pellet was resuspended in fresh spheroplast forming solution. Examination by confocal fluorescence microscopy (see Supplementary Figure 2B) confirmed that both the supernatant and the resuspended pellet after the last centrifugation were free of microspheres. After the 4^th^ centrifugation the pellet was resuspended in rich growth medium with 15% sucrose and incubated for 8 days.

After the incubation both samples were examined by DIC microscopy (Supplementary Figure 2D and E).

### FLIP of giant rod *Hbt. salinarum* cells

**Figure 6:**
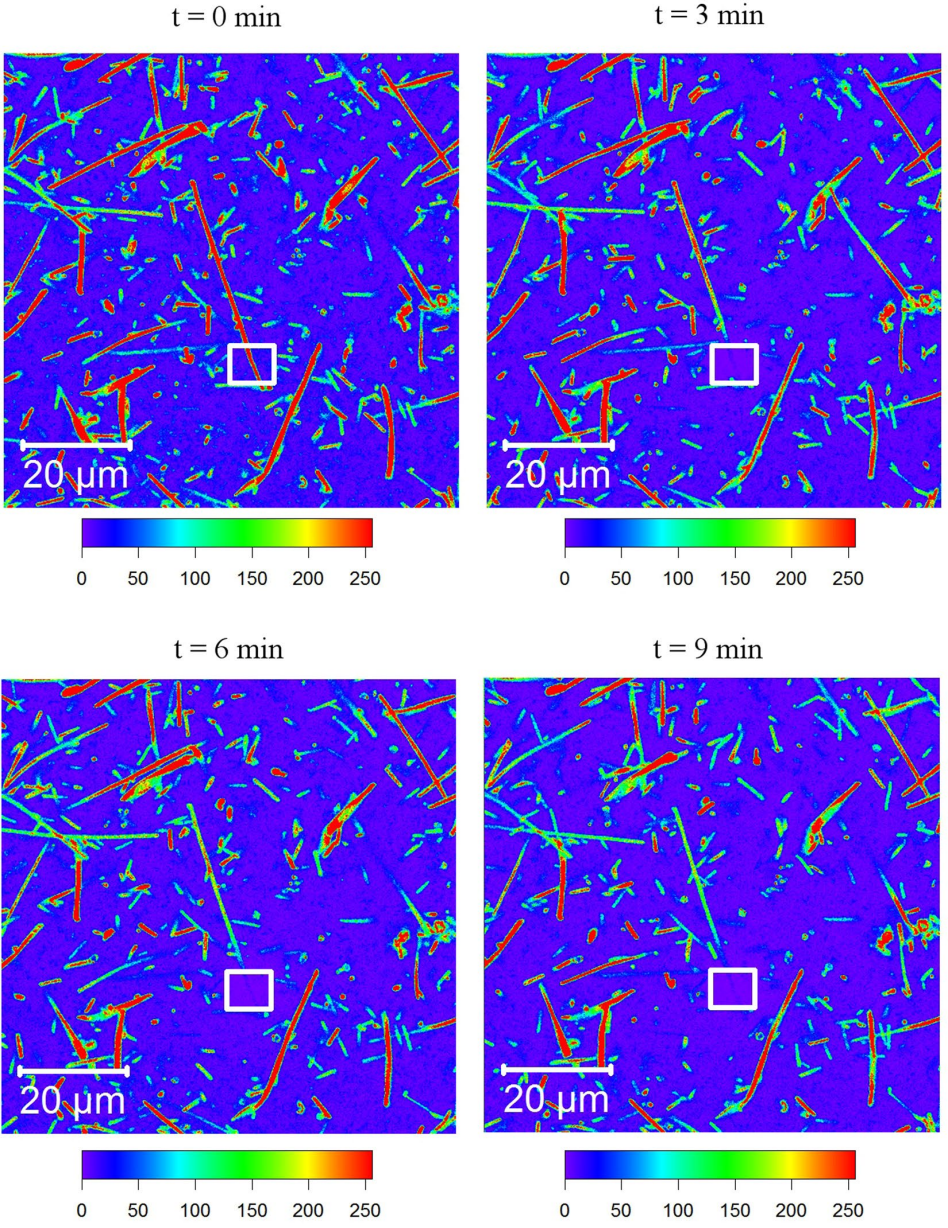
Bleaching of stained giant rod *Hbt. salinarum* cells. The cytosol integrity of the cells (approximately 40 µm long in this case) was demonstrated using FLIP of MitoTracker Orange CMTMRos in *Hbt. salinarum* cells. The bleaching region is marked with a white square, the intensity of the fluorescence signal is shown as a heatmap (pseudocolour, intensity increases from purple to red according to the scale below image). The loss of fluorescence intensity along the entire length of the cell shows that the majority of the dye can freely diffuse along the cell, demonstrating the existence of one cellular compartment not separated by septal membranes.

After 15–20 days of incubation at growth conditions *Hbt. salinarum* cells were stained with MitoTracker Orange CMTMRos and then incubated for several (1–4) days without stirring at room temperature. Rod-shaped cells longer than 30 µm were observed by fluorescence microscopy (see Fig. 6 and Supplementary Video 4) and iteratively bleached (several seconds each cycle) using a high-intensity (100% power) 561-nm laser in the region (typically, 10 × 10 µm^2^) close to one of the rod’s ends, in intervals of approximately 30 s.

### Isolates from environmental samples

Four samples of extremely halophilic microorganisms were isolated from various hypersaline lakes using standard microbiological techniques^55^ and then identified as follows: *Halorubrum sp*. – euryarchaeota from the Alikes salt lake (Kos island, Greece); *Halomonas sp*. and *Haloferax sp*. – proteobacteria and euryarchaeota from the Elton salt lake (Volgograd region, Russia), respectively; and *Salicola sp*. – proteobacteria from the Chott el Djerid salt lake (Tunisia).

The cultures were cultivated in a nutrient medium composed of 250 g/L NaCl, 20 g/L MgSO_4_·7H_2_O, 3 g/L KCl, 3 g/L Na_3_C_6_H_5_O_7_, 0.2 g/L CaCl_2_, 5.0 g/L tryptone (Serva, Germany, 48647.01), 2.0 g/L yeast extract (Organotechnie, France, 19512), 1.25 g/L glycerol, 1 mg/L ZnSO_4_·7H_2_O, 50 mg/L FeSO_4_·7H_2_O,0.3 mg/L MnSO_4_·H_2_O, pH 7.0. The medium was sterilized at 121 °C for 30 min.

Cultivation in liquid medium was carried out at 38.5 °C and 150 rpm using a Unimax 2010 orbital platform shaker (Heidolph, Germany) with illumination (Philips TL-D 18W/33–640 lamps) for 7 days in 100 mL conical flasks with 5 mL of inoculum added to each flask.

The halophilic microorganisms from the environmental samples were identified by comparative phylogenetic analysis of their 16S rRNA gene sequences. For this purpose DNA was extracted using the Wizard technique combined with the modified Birnboim-Doli method^56^. The concentration of DNA was 30–50 µg/mL, RNA was present in trace quantities (<1 %).

A universal primer system (Univ11f-Univ1492r for bacteria and 8fa-1492r for archaea; Evrogen, Russia) was used for PCR and further sequencing of PCR-fragments of 16S rRNA genes^57,58^. PCR products were purified by electrophoresis in a 1% agarose gel followed by extraction with the Wizard PCR Preps DNA Purification System (Promega, Madison, WI, USA) as described in the manufacturer’s protocol.

PCR-fragments of 16S rRNA genes were sequenced by Sanger’s method using a Big Dye Terminator v.3.1 reagent kit (Applied Biosystems, Inc., USA) on an ABI PRIZM 3730 system (Applied Biosystems, Inc., USA) according to standard instructions from the manufacturer. Sequence similarities were identified in BLAST^59^.

16S rRNA sequences were deposited to the GenBank database (MF148853 – *Haloferax sp*., KY781161 –*Halorubrum sp*., KY781162 – *Halomonas sp*., KY781163 – *Salicola sp*.).

## Results and Discussion

### Staining of *Hbt. salinarum* under growth conditions

To find an appropriate method to stain *Hbt. salinarum* we started with the idea that an important intrinsic property of a halobacterial cell is its negative membrane potential. This led us to try to stain halobacteria with MitoTracker cationic probes, which are normally used to stain mitochondria. It is the mitochondrial membrane potential which drives cationic MitoTrackers^60^ into organelles. Halobacteria have membrane potentials of the same sign and can potentially be stained using the same approach.

Indeed, our first experiments showed that living *Hbt. salinarum* cells can be stained in their growth medium using three MitoTracker dyes (MitoTracker Orange CMTMRos, MitoTracker Red-CMXRos, and MitoTracker Deep Red FM), as described in Materials and Methods and observed using a confocal fluorescence microscope (see Fig. 1). Fluorescence signal accumulated from cells is much higher than the background level (SNR >100) and it can be inferred that free dye is drawn into the cells. Apparently, MitoTrackers are driven by membrane potential in the same way as in mitochondria.

We have also tried staining halobacteria with MitoTracker Green FM, which has been shown to stain mitochondria regardless of membrane potential^61^ (data not shown). In this case, no accumulation of the dye in the cells was observed. This additionally supports our hypothesis that membrane potential plays a crucial role in the staining of halophiles with MitoTracker dyes.

### Effects on growth rate and staining durability

To determine whether staining has a negative effect on growth rate, we studied cells stained with MitoTracker Orange CMTMRos (see Materials and Methods for details). Figure 2 shows the growth curve of the stained culture compared with control non-stained one. MitoTracker staining does not alter the normal cell growth rate (with doubling time in the range of 1.2 ± 0.3 days). Cell size and shape remain for at least three generations as observed with a fluorescence microscope (data not shown).

The absence of an effect of MitoTracker dyes on growth rate is highly desirable for a wide range of biological studies and is not shared by the most popular staining alternatives. LIVE&DEAD kit staining is the only approach that was systematically considered in terms of its applicability to living halophiles in their native environment^35^. One of the two examined cell lines (*Halobacterium sp. NRC-1*) exhibited a two-fold decline in CFU (colony forming units) in the presence of the staining reagent^35^. For the other line (*H. dombrowskii H4*), LIVE&DEAD staining has been shown to have almost no effect on cell viability^35^. However, in both cases cells incubated with dyes for long and short time periods were compared, and therefore, a rapid response occurring immediately after staining cannot be excluded and therefore can be a substantial experimental limitation.

Another beneficial property of MitoTracker staining is its durability. The staining persists during washing (removal of supernatant), dilution of stained cells in a MitoTracker-free medium and cultivation of cells (see Materials and Methods, MitoTracker durability during cultivation). As depicted in Fig. 3, the staining quality of *Hbt. salinarum* cells remains satisfactory and provides cell fluorescence well above background through at least 3 cycles of incubation/dilution.

### Conversion of *Hbt. salinarum* to spheroplasts

To validate the staining quality, imaging of a well-known process of halobacterial cell wall removal by EDTA was carried out. In this process, rod-shaped halobacteria stained with MitoTracker Orange CMTMRos were converted to spherical particles called spheroplasts. After several minutes of incubation in a 10 mM EDTA-containing buffer, rod-shaped cells were converted to spheroplasts. The conversion process is depicted in Fig. 4 and Supplementary Video 1.

The shorter individual cells swell at one end (Fig. 4B) and the remaining rod-like part of the bacterium merges with this roundish end-structure (Supplementary Video 1). In this process, cells undergo the same conversion steps as previously described based on phase-contrast microscopy of *Hbt. salinarum*^62^. The longer cells bend around their midpoint in the beginning and then undergo the same process (Fig. 4C). The resulting spheroplasts are clearly visible with SNR >100. Each rod gives rise to one or two spheroplasts depending on its length. In previous studies concerned with osmotic conversions^42,62,63^ the average number of spheres produced (observed by phase contrast microscopy^62,63^, electron microscopy^42^ or fluorescence microscopy based on LIVE&DEAD staining^42^) varied from one to four, which provides evidence that different protocols (salinity change, pH change, EDTA, other cell culture conditions before conversion etc.) can result in different conversion physiology.

In addition to MitoTracker Orange CMTMRos, MitoTracker Red-CMXRos and MitoTracker Deep Red FM were used to study the conversion process, which resulted in similar observations. The quality of final spheroplast staining was equally good for all three MitoTracker dyes and is shown in Supplementary Figure 1.

### Microspheres formed during spheroplast conversion

Recently, it was shown that rod-shaped Halobacterium species can convert to microspheres (approximately 0.4 µm in diameter) upon a decrease in water activity below 0.75^42^. The term water activity is widely used to define the availability of water for hydration of materials. A value of 1.0 indicates pure water, 0.75 approximately corresponds to 4 M NaCl buffer. One rod forms three to four microspheres that remain viable for years and proliferate into normal rods when supplied with proper nutrients. Microspheres with similar properties form in fluid inclusions in laboratory-grown halites as well as in salt deposits isolated from millions-year-old minerals. Presumably, microspheres represent a dormant form of haloarchaea with higher resilience, which allows them to survive in unfavourable environmental conditions^22^.

Surprisingly, the MitoTracker staining approach revealed similar microspheres to coexist with spheroplasts after the EDTA conversion (see Supplementary Figure 2A) according to the standard protocol described in Materials and Methods (Conversion to spheroplasts). To identify the origin of the microspheres, we recorded the conversion of *Hbt. salinarum* to spheroplasts, trapped in a 2% agarose gel (see Supplementary Video 2 and Fig. 5). Single microspheres are formed from some halobacteria while they lose their S-layer during spheroplast conversion. Restoration of rod-shaped *Hbt. salinarum* from spheroplast solution has been well described in previous studies and is widely used for *Hbt. salinarum* transfection^46,64^. Since microspheres were not previously observed in a spheroplast solution, the question arises whether restored rod-shaped *Hbt. salinarum* cells originate from microspheres, spheroplasts or both. To tackle this question we separated spheroplasts and microspheres as described in Materials and Methods (Recovery of rod-shaped Hbt. salinarum from spheroplasts and microspheres). Interestingly, after cultivation for 10 days in nutrient-rich medium, both samples restored rod-shaped halobacteria species (see Supplementary Figure 2D and E). This result implies that microspheres formed from halobacteria during spheroplast conversion possess all that is necessary for growing into normal rod-shaped halobacteria. Similar to the case of fluid inclusion, microspheres may serve as a halobacterial back-up to thrive in unfavourable conditions.

### Giant rod *Hbt. salinarum* cells

Most *Hbt. salinarum* cells observed in our experiments are rod-shaped with an approximate length of 9 µm (see Supplementary Video 3). Мuch longer rod-shaped cells were occasionally found with a length of up to 45 µm. Notably, the length of a cell correlated with its motility: longer cells were less motile. The fraction of giant rod cells was higher when cells were incubated for a long time in a stationary phase without dilution (15–20 days). These observations are in good agreement with earlier data showing long and immobile *Hbt. salinarum* cells in surface-adherent biofilms (see Fig. 1 in^65^ and Fig. 5 in^43^).

Little is known about the morphology of giant rod halobacterial cells. We observed that long rod cells split into several smaller ones during spheroplast formation (Fig. 4 and in Supplementary Video 1). Thus, it could have been expected that giant rod cells consist of several smaller individual cells that remain concatenated after division. To test whether the giant rods are single cells and whether their cytosols are not divided into sub-compartments, we investigated the diffusion of MitoTracker dyes inside single giant rod cells. As described in greater detail in Materials and Methods (FLIP of the giant rod *Hbt. salinarum* cells), we bleached the MitoTracker dye on one end of the giant rod stepwise, and recorded the reduced fluorescence intensity along the whole cell (Fig. 6 and Supplementary Video 4). Notably, the fluorescent dye is not completely bleached using this approach, which shows that a fraction of the dye does not diffuse freely but remains attached to some cell compartments. The binding of the dye’s chloromethyl group to thiol groups of immobile intracellular (or membrane-associated) proteins is a likely explanation^61^.

### Isolates from environmental samples

**Figure 7:**
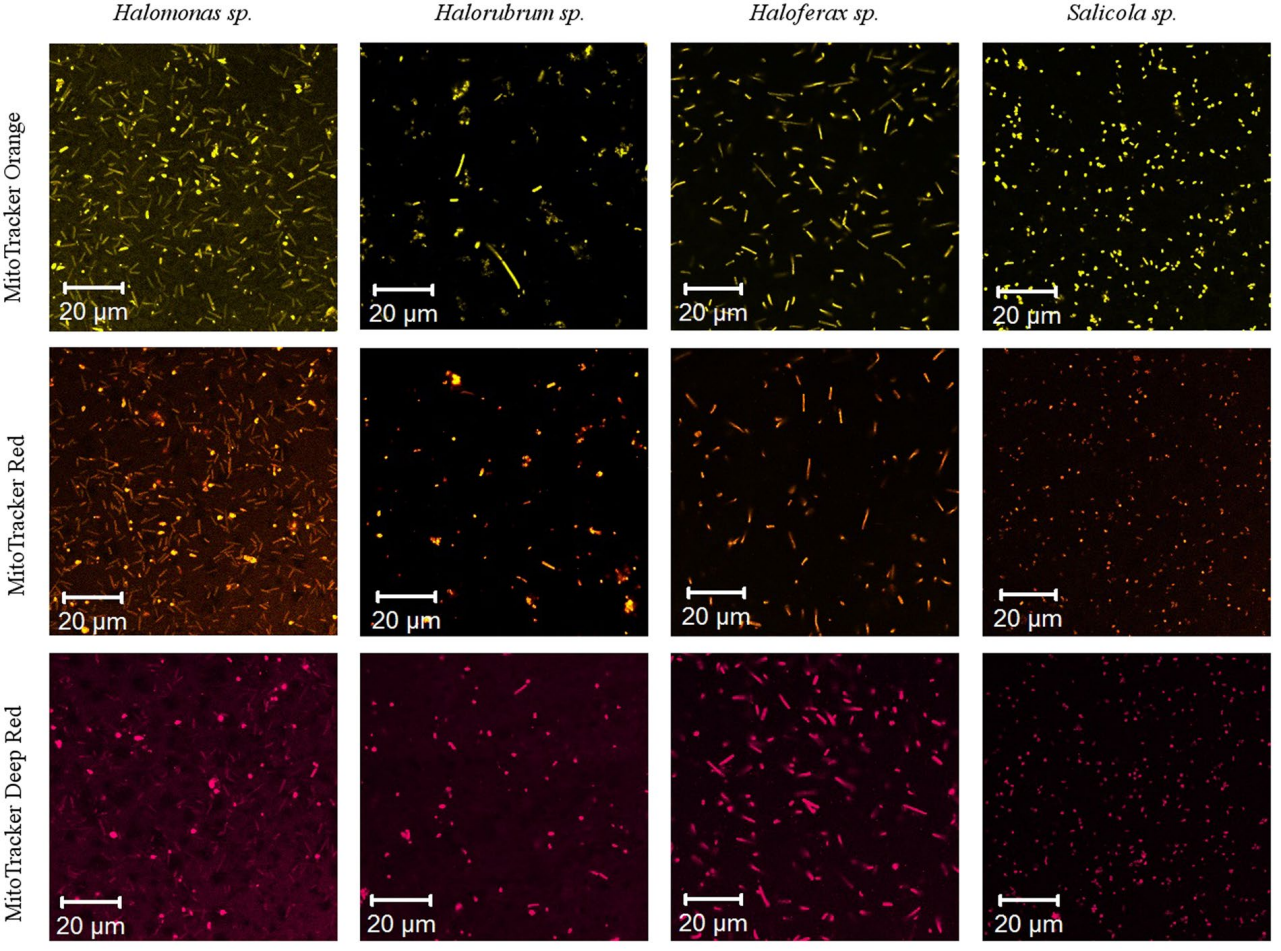
MitoTracker dyes staining of halophilic microorganisms (*Halomonas sp., Halorubrum sp., Haloferax sp., Salicola sp*.) isolated from hypersaline lakes.

To check the general applicability of our MitoTracker dye staining approach for other halophile species we tested four strains of microorganisms, isolated from environmental samples from hypersaline lakes: *Halorubrum sp*. – euryarchaeota from the Alikes salt lake at the Kos island, Greece; *Halomonas sp*. and *Haloferax sp*. – proteobacteria and euryarchaeota from the Elton salt lake, Volgograd region, Russia; and *Salicola sp*. – proteobacteria from the Chott el Djerid salt lake, Tunisia. These selected halophile species belong to different domains of life: 2 are bacteria and 2 are archaea. The samples were stained with three different MitoTracker dyes, similar to *Hbt. salinarum*, and were examined using a confocal fluorescence microscope. In all cases, samples showed bright homogeneous staining of cells, as expected (see Fig. 7). This success provides evidence that the staining mechanism is general and not restricted to *Hbt. salinarum* or even to a single domain of life. Thus the suggested staining technique can be applied for the detection and analysis of yet unknown microorganisms in extremely halophilic environments or in extraterrestrial samples.

## Conclusions

MitoTracker dyes of three different colours (MitoTracker Orange CMTMRos, MitoTracker Red CMXRos, MitoTracker Deep Red FM) were shown to stain five halophilic microorganisms (three archaea and two bacteria) under growth conditions with high staining efficiency and low residual free dye level in the medium. The staining procedure does not require a washing step.

Staining quality of MitoTrackers is sufficiently good for studying the morphology of halophiles. The high SNR is suitable for morphological studies, cell counting and detection, which is important for future applications. Staining persists during the harsh spheroplasts conversion protocol, which implies that MitoTracker dyes can be used even when severe washing procedures are required. The MitoTracker dye staining does not affect growth rate and is inherited through several cell generations, making it a good choice for long-term studies, which require non-cytotoxic staining of live cells.

Our observations suggest that MitoTracker dyes are drawn into the cell by the membrane potential. Subsequently, they become anchored inside the bacteria via chloromethyl group binding to thiol groups of intracellular proteins as was previously described for MitoTrackers^66^.

Overall, MitoTracker dyes are an ideal choice for staining of halophiles and have many advantages compared with the previously applied staining techniques. Using this new approach, we demonstrated the formation of viable microspheres during *Hbt. salinarum* spheroplast conversion, and cytoplasmic continuity of giant rod *Hbt. salinarum* species.

## Electronic supplementary material

Supplementary video 1

Supplementary video 2

Supplementary video 3

Supplementary video 4

Supplementary Information
