# Supplementary material for: Efficient non-cytotoxic fluorescent staining of halophiles
Source: Sci Rep. 2018 Feb 7;8:2549. doi: 10.1038/s41598-018-20839-7 (PMC5803262; doi:10.1038/s41598-018-20839-7)
Supplement: Supplementary file 5 — Supplementary Information [file 41598_2018_20839_MOESM5_ESM.pdf]

SUPPLEMENTARY INFORMATION

Efficient non-cytotoxic fluorescent staining of halophiles

Ivan Maslov[1], Andrey Bogorodskiy[1], Alexey Mishin[1], Ivan Okhrimenko[1], Ivan Gushchin[1], Sergei Kalenov[2], Norbert A. Dencher[1,3], Christoph Fahlke[4,1], Georg Büldt[1], Valentin Gordeliy[1,5,6], Thomas Gensch[4], and Valentin Borshchevskiy[1]*

[1] Research Center for Molecular Mechanisms of Aging and Age-Related Diseases, Moscow Institute of Physics and Technology, 141700, Dolgoprudniy, Russia

[2] Mendeleyev University of Chemical Technology of Russia, 125047, Moscow, Russia

[3] CSI Organic Chemistry and Biochemistry, Technische Universität Darmstadt, 64287 Darmstadt, Germany.

[4] Institute of Complex Systems (ICS), ICS-4: Cellular Biophysics, Forschungszentrum Jülich GmbH, 52428 Jülich, Germany

[5] Univ. Grenoble Alpes, CEA, CNRS, IBS, 38000 Grenoble, France

[6] Institute of Complex Systems (ICS), ICS-6: Structural Biochemistry, Forschungszentrum Jülich GmbH, 52425 Jülich, Germany

* borshchevskiy.vi@phystech.edu

Supplementary video 1.

EDTA-conversion of *Hbt. salinarum* cells stained with MitoTracker Orange CMTMRos to spheroplasts. The events of conversion are highlighted using purple circles: the small rods become bulgy, beginning at one end. The longer cells first bend around the midpoint and then convert to spheroplasts. The conversion process depicted here has a real-time duration of 10 min.

Supplementary video 2.

Formation of microspheres during EDTA-conversion of *Hbt. salinarum* cells to spheroplasts. *Hbt. salinarum* cells stained with MitoTracker Orange CMTMRos were entrapped in a 2% agarose gel and incubated in an excess of a spheroplast-forming solution with 50 mM EDTA at 37°C until conversion was detected via fluorescence microscopy. In addition to spheroplasts the smaller spherical objects, here called microspheres, appear during the conversion (highlighted using purple circles). In contrast to rod-shaped cells and spheroplasts, the microspheres are mobile in agar, but fluctuate only in the proximity of spheroplasts that originated from the same rod-shaped cells. This can be explained by fluctuation inside cavities formed in the agar after the rod-shaped cells contracted. The conversion to spheroplasts and microspheres depicted here lasts 1 hour in real time. The video was processed for drift correction using the free plugin “StackReg” for Fiji.

Supplementary video 3.

Giant rod *Hbt. salinarum* cells revealed via MitoTracker Orange CMTMRos staining. The cells stained with MitoTracker Orange CMTMRos were observed using fluorescence microscopy in the growth medium. While the majority of cells are very mobile and have lengths of appx. 9 µm, there is a subpopulation of longer (here approximately 30 µm) and relatively immobile cells (called “giant rod cells”). The fraction of long cells is increased in the depicted sample using long (15 days) incubation of cell culture in a stationary growth phase before staining without dilution or medium change. The video sequence depicting the motility of *Hbt. salinarum* cells lasts 7 min in real time.

Supplementary video 4.

Bleaching of giant rod *Hbt. salinarum* cells stained with MitoTracker Orange CMTMRos. The cells were incubated in growth medium at 37°C in an incubator compatible with a fluorescence microscope in the dark for 24 hours to reduce cell motility. Then, the integrity of giant rod cell’s cytosol was examined using the FLIP (Fluorescence Loss In Photobleaching) method: the dye was bleached using a 561-nm laser at one end of the rod, which resulted in a decrease in fluorescence intensity along the entire -giant rod cell. The shown photobleaching sequence is 17 min long.

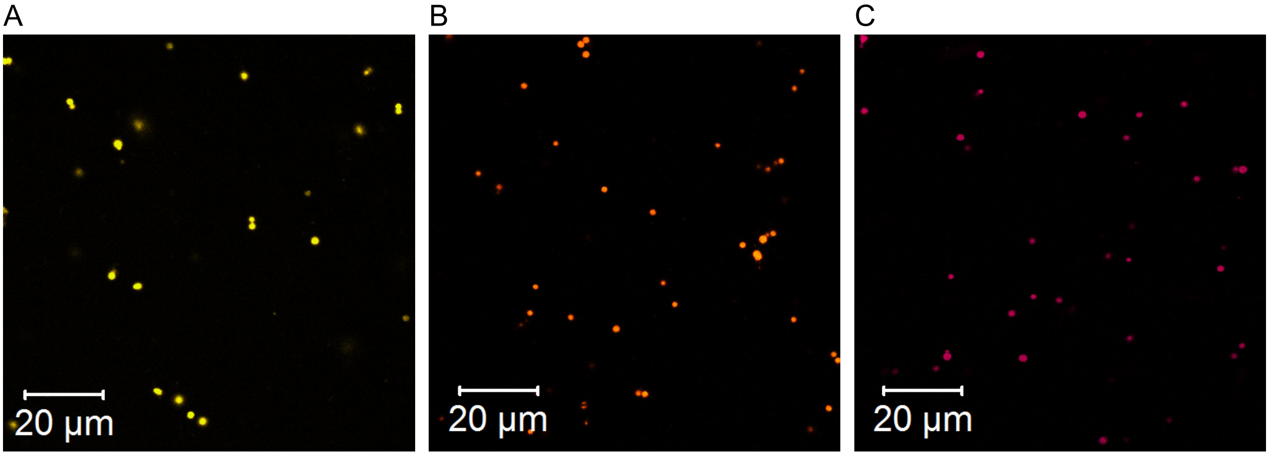

Supplementary figure 1. Hbt. salinarum spheroplasts stained with (A) MitoTracker Orange CMTMRos (M7510), (B) MitoTracker Red CMXROS (M7512), (C) MitoTracker Deep Red FM (M22426). Staining was performed before the conversion to spheroplasts.

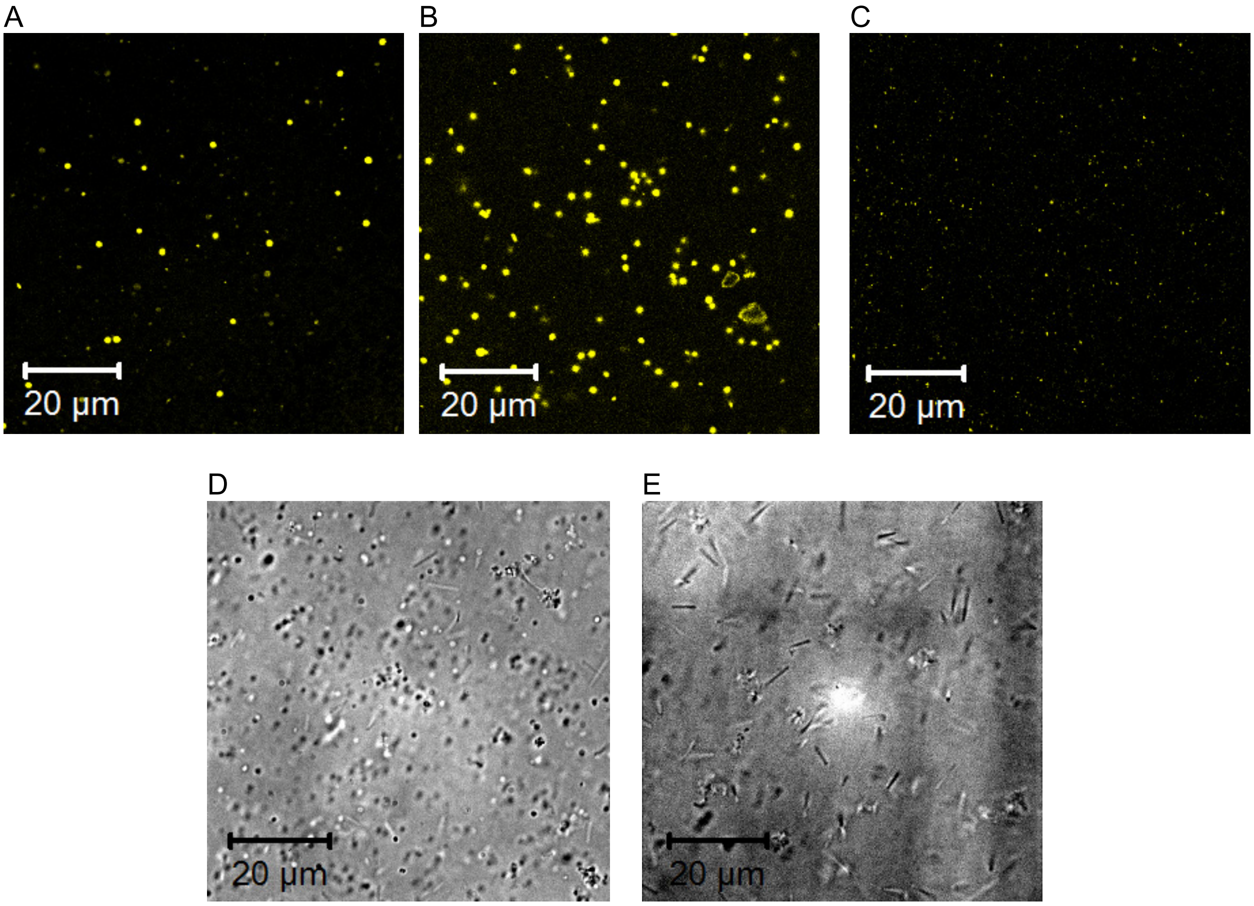

Supplementary figure 2. Spheroplasts and microspheres isolated from the EDTA-converted *Hbt. salinarum* cells, and the rod-shaped cells recovered after incubation in a nutrient medium at growth conditions. A. Initial mixture after the EDTA conversion. B. Spheroplasts isolated by centrifugation. C. Microspheres isolated by filtration through a 0.45-µm PTFE filter (see materials and Methods, Recovery of rod-shaped Hbt. salinarum from spheroplasts and microspheres). D, E. DIC images of rod-shape cells recovered from spheroplasts and microspheres, respectively.
